# Diseases of the Circulatory System

**Published:** 1905-09-23

**Authors:** 


### Progress in Medicine and Surgery.

### DISEASES OF THE CIRCULATORY SYSTEM.

**The Use of Venesection.**—Recent researches by Hyrtl have thrown some light on the viscosity of the blood, and enable us to measure the great reduction in the work of the heart which would take place if the vessels were filled with saline fluid in place of blood. Now it is well known that after bleeding the volume of the blood is quickly made up by absorption of lymph. This is equivalent to dilution and a reduction of the viscosity. Ewart has investigated the utility of bleeding in the light of these facts.[1] He points out that the circulatory system is a self-regulating machine for distributing the red corpuscles with their loads of oxygen for the needs of the tissues. It appears that in order to get the greatest number of ordinary blood corpuscles through the capillaries with the least expenditure of energy, the blood should contain a certain number of them per cubic millimètre. A greater or smaller density involves more work. This ideal density turns out to be actually the one existing in normal blood. If, then, there are less than 5,000,000 red cells the gain from the diminished viscosity does not compensate for the loss of carrying power, and the heart, to supply the needs of the tissues, has to expend more energy and to raise the tension. This high tension actually occurs in anæmia, provided that the cardiac muscle is intact. On the other hand, if the corpuscles are more than 5,000,000 per cubic millimètre, the heart, to keep up normal oxygenation, must do more work, or the capillaries must dilate, and in either case, if the vessels are diseased, rupture of a vessel may take place. The bearing of all this on the effects of venesection is clear. If anæmia exists, bleeding is harmful, for it results in a further dilution of the blood and increased work for the heart. The best cardiac tonic here is a hæmatinic. If there is an excess of red cells, venesection is useful and relieves the heart. In pneumonia if much inflammatory exudation occurs the blood may become concentrated, but the pyrexia often has a compensatory action. If cardiac distress, however, comes on, venesection is useful provided that the heart has still power to regain lymph to replace it. This condition only exists at an early stage of the disease. In asphyxia the increased density of the blood may increase the difficulties of the heart, and here therefore venesection is sometimes of value. When anæsthetics are badly borne, the withdrawal of a little blood improves the circulation. Hence the improvement sometimes seen after an operation has begun. These investigations tend to show that venesection is not often useful. The conditions in which there is an excess of corpuscles are not common, and where the latter are deficient bleeding is harmful. The temporary fall of pressure which follows copious hæmorrhage may occasionally be desired for some special purpose, but the other remedial results of bleeding are only obtained under certain conditions. We may also refer to the compensatory high tension which is said to occur in cases of compression of the brain. Bleeding has been often recommended here, and certainly the highest pressures known are found in such cases, but some investigations tend to show that the tension is an effort of nature to keep the vital centres supplied with blood under the special difficulties, and should not be interfered with except by removing the cause, such as a depressed cranial fracture. If the cause is a cerebral hæmorrhage which compresses the brain, and is not of traumatic origin, but is itself due to hypertension, the question of treatment by bleeding and purgation becomes a difficult one, and on the other hand the operation of trephining and removing the clot is usually hopeless.

**Failure of Myocardial Conductivity.**—The diagnosis of this condition is shown to be possible by J. Mackenzie.[2] Certain obscure forms of heart failure turn out to be cases of this kind, and can be determined by the use of a double sphygmograph giving simultaneous records of the jugular and carotid pulses. Where the conduction of the systolic wave is hindered, the interval between the two pulses becomes prolonged and will exceed one-fifth of a second. The cardiac irregularity is made worse by digitalis, and under its influence many waves never reach the ventricle, so that we get coupled beats. The drug indeed seems easily to affect conduction of the waves, for even in normal hearts excessive doses produce the same phenomenon of coupled beats. Another form of heart failure with dilatation, continuous irregularity, or paroxysmal tachycardia, and generally no valvular lesion seems to be due to the wave commencing at the auriculo-ventricular sinus instead of at the sinus venosus. Here the jugular pulse tracing ceases to have its normal form and gets that of an arterial one. Unfortunately this excessive irritability of the fibres about the first-named sinus is not easily overcome by digitalis or any known treatment and may persist for years. Mackenzie's comparison of tracings from the auricles and ventricles is certainly of great interest, and even if we do not accept all his conclusions, we cannot but be grateful for the light his researches throw on certain obstinate cases of irregular heart not infrequently met with, where digitalis and ordinary drugs fail entirely without any obvious reason.

**The Detection of Aortic Stenosis.**—Some writers consider this a common lesion and many people are apt to put down any systolic murmur heard in the neighbourhood of the aortic valve to obstruction of that orifice, but there is strong evidence to show that this is far from correct; for after death it is comparatively rarely found. Again, the lesion has been described as most common in early life, but as a matter of fact though it may arise from acute endocarditis it is more often due to slow sclerosis in the latter part of a laborious life. F. W. Price[3] remarks that we must not diagnose stenosis, unless the typical slow regular and small pulse is present as well as the murmur and after some time hypertrophy of the ventricle. Moreover the second sound is lost or diminished, and there may be a thrill over the base or over the whole precordia. What, then, are the conditions which we may easily mistake for aortic stenosis. Price enumerates (1) cardio-pulmonary murmurs, which have the peculiarity of disappearing after a deep inspiration; (2) hæmic bruits; (3) pulmonary obstruction, but this murmur is never carried up the vessels of the neck; (4) mediastinal tumours producing bruits by pressure, but here there is usually an area of dulness and no alteration of the second sound; (5) mere roughening of the orifice or dilatation of the aorta; but here, too, the second sound is accentuated, and in dilatation is widely audible. On the other hand, in true aortic stenosis this sound is enfeebled or lost, unless there is arterio-sclerosis. Finally, if a double murmur occurs with definite regurgitation we cannot say that stenosis exists so long as the water-hammer pulse is present. With such a number of conditions in which a systolic murmur can be heard there is no reason for surprise that aortic stenosis has been supposed to exist much oftener than post-mortem records show to be the fact. The question of treatment cannot be discussed at length. There is unfortunately little to be done except to prevent any tendency to anæmia and to high vascular tension. Thus Price speaks of the importance of giving rest as much as possible to the crippled organ. Among drugs some relief may be obtained by the use of the iodides and of nitrite of soda. It is curious to compare with this lesion tricuspid obstruction. J. M. Patton[4] finds the latter more common than is supposed. It is especially frequent in females in the third decade of life. Like aortic trouble it may arise from acute rheumatic inflammation or from chronic changes due to over-exertion. As it is in most instances associated with mitral lesions, the symptoms of the pure disorder are seldom met with. These are a pre-systolic murmur which is not due to mitral stenosis, and an enlarged right auricle with venous stasis. There is not much tendency to dyspnœa, but there may be a good deal of susceptibility to cold, cyanosis, and possibly venous pulsation.

[1] Med. Chron., April. [2] Brit. Med. Jour., March 18. [3] Clin. Jour., Jan. 4. [4] Med. Rec., April 26.

